# The paradox of retained genetic diversity of *Hippocampus guttulatus* in the face of demographic decline

**DOI:** 10.1038/s41598-021-89708-0

**Published:** 2021-05-17

**Authors:** Rupert Stacy, Jorge Palma, Miguel Correia, Anthony B. Wilson, José Pedro Andrade, Rita Castilho

**Affiliations:** 1 Universidade Do Algarve, Campus de Gambelas, 8005-139 Faro, Portugal; 2 Centre for Marine Sciences, Campus de Gambelas, 8005-139 Faro, Portugal; 3 Department of Biology, Brooklyn College, Brooklyn, NY 11210 USA; 4 The Graduate Center, City University of New York, New York, NY 10016 USA

**Keywords:** Population genetics, Evolutionary biology

## Abstract

Genetic diversity is the raw foundation for evolutionary potential. When genetic diversity is significantly reduced, the risk of extinction is heightened considerably. The long-snouted seahorse (*Hippocampus guttulatus*) is one of two seahorse species occurring in the North-East Atlantic. The population living in the Ria Formosa (South Portugal) declined dramatically between 2001 and 2008, prompting fears of greatly reduced genetic diversity and reduced effective population size, hallmarks of a genetic bottleneck. This study tests these hypotheses using samples from eight microsatellite loci taken from 2001 and 2013, on either side of the 2008 decline. The data suggest that the population has not lost its genetic diversity, and a genetic bottleneck was not detectable. However, overall relatedness increased between 2001 to 2013, leading to questions of future inbreeding. The effective population size has seemingly increased close to the threshold necessary for the population to retain its evolutionary potential, but whether these results have been affected by sample size is not clear. Several explanations are discussed for these unexpected results, such as gene flow, local decline due to dispersal to other areas of the Ria Formosa, and the potential that the duration of the demographic decline too short to record changes in the genetic diversity. Given the results presented here and recent evidence of a second population decline, the precise estimation of both gene flow and effective population size via more extensive genetic screening will be critical to effective population management.

## Introduction

The loss of genetic diversity is a primary concern in conservation biology and should be at the forefront of strategy to promote biodiversity conservation management^1,2^. It was once thought that marine fishes were too abundant and fecund to be faced with threats from overexploitation^3^. Fishing was a right, oceans were open access, and baseline data were, more or less, non-existent. More than a century of literature and observations later, it is now recognized that the paradigm of inexhaustible marine fishes and exploitation is unequivocally false^4–6^. Unfortunately, while the burden of proof often falls to scientists to demonstrate that anthropogenic activity is the root cause of environmental degradation, the lack of baseline data for comparison often hampers inferences on impact^7^. As a result, many marine environments have been subjected to degradation in the form of pollution, overexploitation and habitat loss. While the paradigm of inexhaustible ocean resources has eroded, many ongoing discussions continue in the field of conservation biology. One of which, relevant here, was proposed by Lande^8^, who suggested that species are often driven to extinction before genetic consequences have time to take effect. However, a bulk of empirical evidence indicates the contrary^9^, and more recently, reduced genetic diversity has been documented in threatened species^10^. Quantifying gene flow, metapopulation structure and demographic and stochastic events are necessary when assessing whether a reduction in population size is likely to lead to a reduction in genetic diversity^11^.

Interestingly, just as there has been contention about the effects of genetic changes on threatened populations, a similar debate has been held over inbreeding in the wild. Despite many questioning the likelihood that inbreeding depression can lead to declines in wild populations^12–14^ that ca. 90% of studies have shown reduced fitness in inbred populations compared with non-inbred individuals^15^. For example, inbreeding depression was reported in 99 species of birds in which failed hatching rate increased with genetically similar parents^16^, as well as in fish, where inbred populations of *Poeciliopsis monacha* suffered from spinal curvature, deformation and reduced resistance to low oxygen^17^.

While inbreeding depression may not inevitably lead to population decline, its interaction with basic parameters of a population’s viability, such as population growth rate and variation in population size, may influence population persistence. A population may experience positive growth and reach its carrying capacity, but when hampered with sequential levels of inbreeding, may reach that carrying capacity at a slower rate. Should a population reach a relatively high level of inbreeding, the negative effect of inbreeding depression may cause population growth to become negative, setting it on a path to possible extinction^18^. This is why maintaining genetic diversity in a population is so important. If evolutionary potential reflects the capacity of a population to adapt to environmental change, then genetic diversity is the raw material that enables natural selection to select for adaptations that are beneficial for dealing with those environmental changes^18,19^. Additionally, population genetic theory predicts that populations with small effective population size (*N*_*e*_) lose genetic diversity faster than populations with a larger *N*_*e*_ due to genetic drift^20^. Thus, genetic factors are important to consider when evaluating extinction risks because threatened species often have smaller and/or declining population sizes^21^. In such populations, the loss of genetic diversity and an increased probability of inbreeding is inevitable^18^.

Seahorses are marine fish with relatively fast growth rates, which mature at young ages, and have short generation times^22^, suggesting that seahorse populations may rapidly recover from population declines^23^. However, their low mobility, monogamous mating pattern, relatively few offspring, mate fidelity and elaborate parental care^22^ could increase their vulnerability.

*Hippocampus guttulatus* Cuvier, 1829, is one of two seahorse species occurring in the north-east Atlantic Ocean from the North African coast to the coast of Shetland Isles, into the North Sea and in the United Kingdom, the Mediterranean and the Black Sea. Along with the sympatric *H. hippocampus*, both species are classified as ‘Data deficient’ at global and regional European levels and near-threatened within the Mediterranean, according to regional assessments from the IUCN^24,25^. The long-snouted seahorse displays a preference for shallow, sheltered and complex coastal habitats, with high seagrass density and extensive vegetation cover that provide holdfasts and abundant food^26,27,37^. It reaches its highest abundances in marine lagoons^27–29^. The species’ dependence on shallow and protected environments that are physically isolated from each other potentially limits its dispersal across unsuitable habitat stretches.

DNA sequence variation in *H. guttulatus* across its range is low (1.23% and 1.49% in mitochondrial cytochrome b and control region, respectively)^30^. Previous genetic differentiation assessments revealed the existence of five cryptic lineages across the species distributional range^31,32^: Parapatric North and South European Atlantic lineages (including Portugal), which meet in Southwest France, where they coexist in sympatry; two lineages in the Mediterranean, associated with lagoon and marine habitats; and a Black Sea lineage^31,32^. More regional studies in the NW Iberian Peninsula based on 13 microsatellite loci found no evidence of reproductive isolation^33^. Recent work on the Mediterranean marine lagoons of Taranto in southern Italy and Thau in the Gulf of Lion, France, based on eight microsatellite loci and a mitochondrial DNA region (cytochrome b) revealed private mitochondrial haplotypes and unique genotypic profiles that make these populations distinct from marine coastal locations^34^. The populations’ low genetic diversities in those two lagoons are consistent with a severe or long bottleneck^34^.

**Figure 1:**
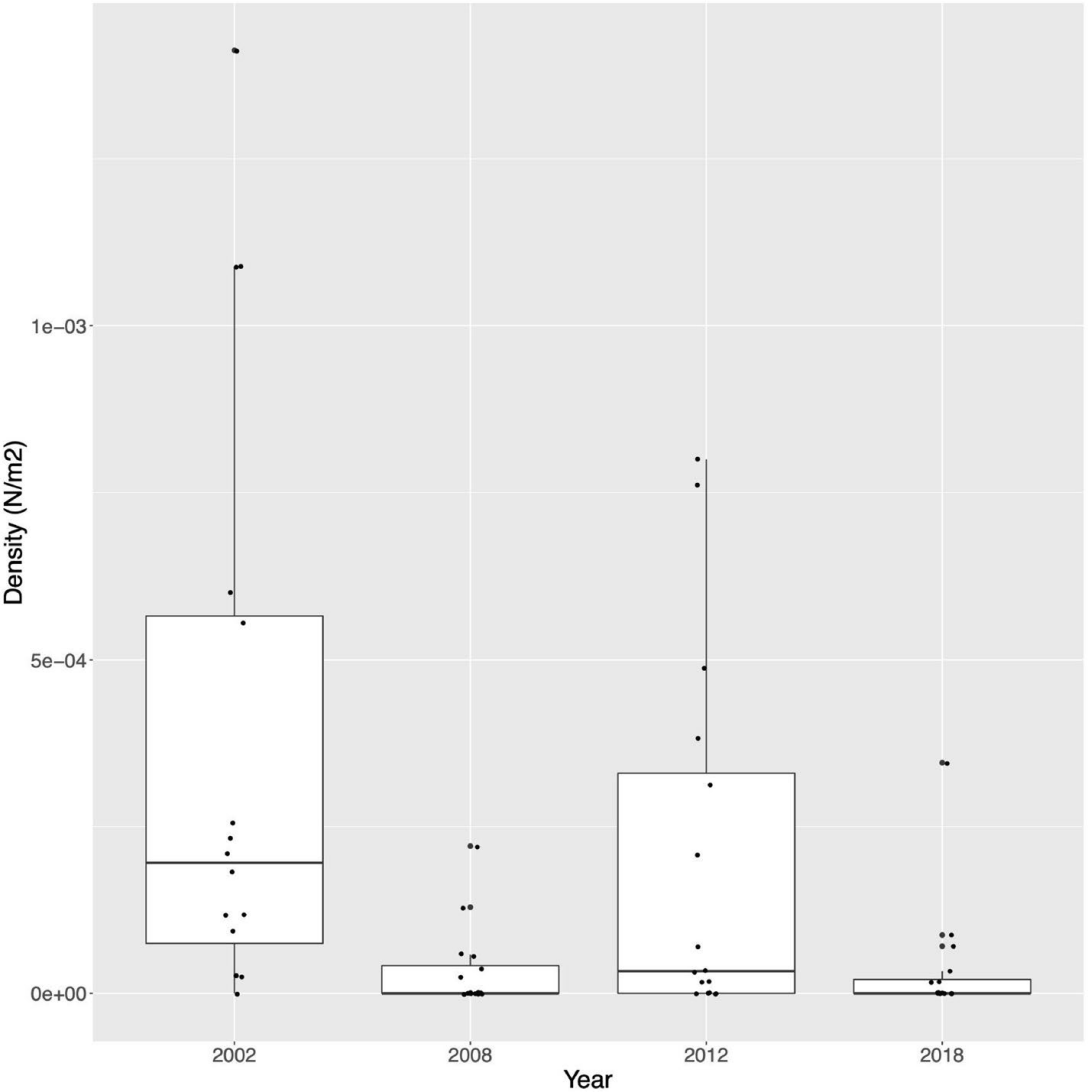
Demographic changes of Ria Formosa’s *H. guttulatus*. Data from^37^, and J. Palma (pers. comm.).

**Figure 2:**
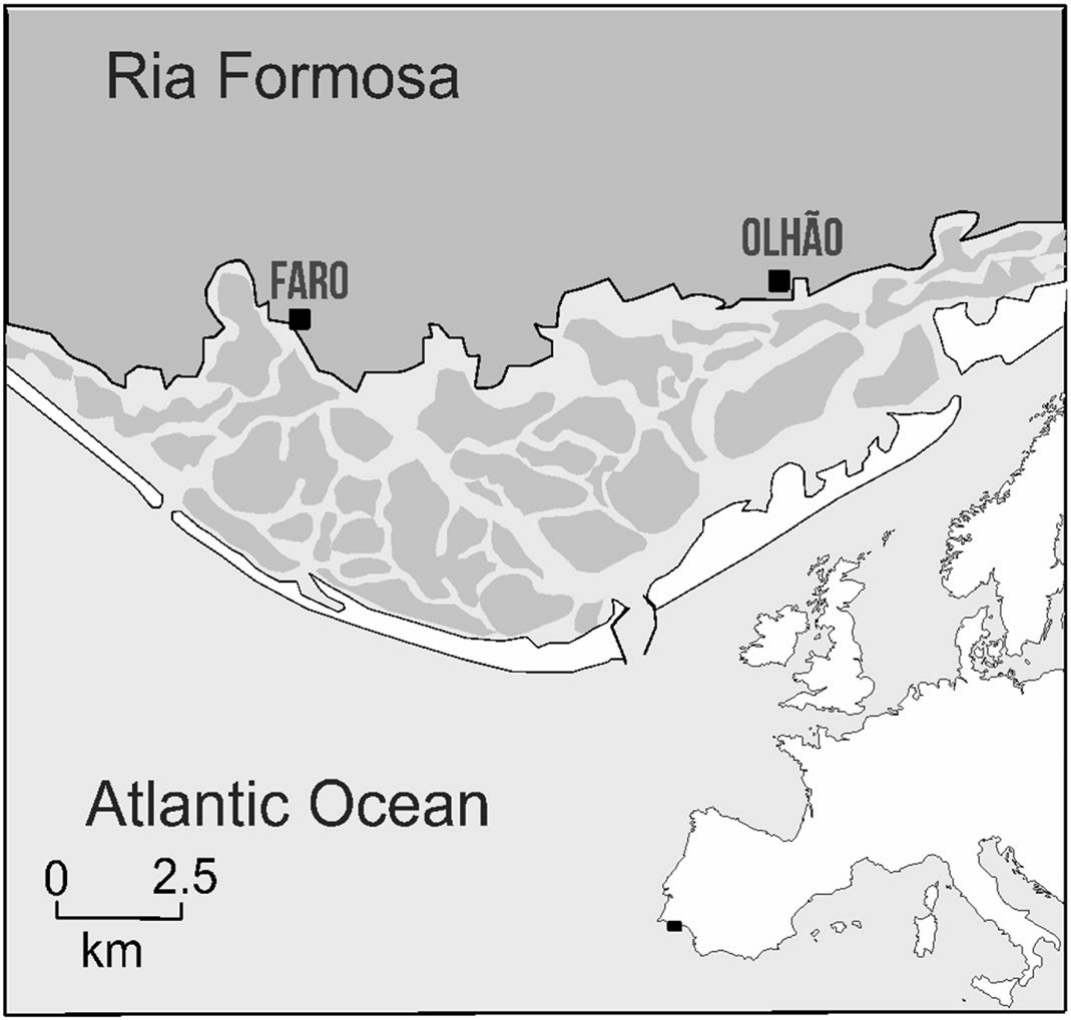
The Ria Formosa barrier island system, South Portugal. The black box on the European map represents the sampling region. The Ria Formosa barrier island system, South Portugal. The black box on the European map represents the sampling region. Raw map was downloaded from https://freevectormaps.com/ and edited in Adobe Illustrator CC2019 (version 23.0.1) (https://www.adobe.com/products/illustrator.html).

The population of *H. guttulatus* living in the Ria Formosa, South Portugal (Fig. 2), was once noted as having particularly high densities, an order of magnitude higher than similar seahorse surveys in other locations of this species’ geographic distribution^26,35^ Woodall et al.^30^. A significant reduction in density from 0.09 n*/*m^−2^ in 2001/2002 to 0.007 n*/*m^−2^ in 2008/2009, representing a 94% decrease, suggested a severe decline of this population. A subsequent rebound to 0.053 n*/*m^-2^ in 2010–2013, no significant differences between the 2001/2002 and the 2010/2013 (2012 in Fig. 1) population surveys, and a positive correlation between density and holdfast availability could indicate an extreme population fluctuation rather than a consistent downward decline^36^. However, more recent surveys suggest similar densities to those detected in 2008/2009 survey, indicating a relapse and an overall decline over ~ 20-year period^37^ (Fig. 1).

While the strong decline in 2008 has been attributed to habitat loss^36–38^, we cannot discard the role of other variables such as warmer temperatures, food availability or abundance of predators^36^. The decline in 2018 is most probably due to illegal fishing (J.Palma, person.comm.). Unfortunately there are no data available on the possible effect of these variations in density over this short temporal window on population dynamics of the species except that more recently only one large class size was observed (J.Palma, pers. obser.).

Extinction rates are estimated to be three to eight times higher for populations than for species^39^. As genetic diversity provides the raw foundation for adaptation and evolutionary potential, acquiring estimates of genetic diversity of the Ria Formosa’s *H. guttulatus* population in the light of extreme fluctuation and continued anthropogenic environmental change would provide critical insights into patterns of demographic change in contemporary time, and suggest potential management responses.

The nature and severity of the threats faced by the *H. guttulatus* population in the Ria Formosa and declining population trends may lead to the suspicion that this population is facing a demographic bottleneck that may induce genetic erosion. Using two genetic snapshots of samples taken 12 years apart, this study aims to examine the effects of population fluctuations as inferred by census data on the genetic diversity of the Ria Formosa *H. guttulatus* population. Individuals were sampled in 2001 and 2013, and for both years estimates of observed and expected heterozygosity, allelic richness, private allele richness, effective population size and relatedness were calculated for comparison. This study hypothesizes that the severe population decline observed in the 2008 survey would likely reduce the effective population size of the following generations and contribute to a reduction in the genetic diversity of *H. guttulatus.*

## Material and methods

### Sampling

Seahorses were individually sampled in 2001 (N = 76) and 2013 (N = 258) from Ria Formosa (Fig. 2) by scuba diving. When a seahorse was found, two skin filaments (4 mm on average) were clipped using sharp scissors, one millimetre above the bone insertion of the filament. The clipped filaments were placed in 15 ml Falcon tubes filled with seawater until the end of the underwater sampling process and transferred to an Eppendorf tube filled with 95% ethanol and kept in the freezer prior to DNA analysis. This non-lethal method does not impact the health and welfare of the animal, as skin filaments rapidly regenerate^40,41^. The sampling procedures comply with the guidelines of the European Union Council (2010/63/EU). All protocols were approved by the ethical committee ORBEA of CCMar / University of Algarve and performed under a ‘‘Group-C” license from the Direcção-Geral de Veterinária, Ministério da Agricultura, do Desenvolvimento Rural e das Pescas, Portugal.

### DNA extraction, amplification, fragment analysis, genotyping and pre-processing

DNA from filament samples was extracted by standard phenol–chloroform protocol, according to Sambrook^42^. Following DNA extraction, amplification of 12 highly polymorphic microsatellite loci (Electronic supplement S1) isolated for *H. guttulatus* (or obtained by cross-amplification in *H. hippocampus*) was performed as described in Pardo et al.^43^ and van de Vliet et al.^44^. PCR reactions with labelled primers were performed using standard procedures, and amplified products were run on an ABI 3130XL (Applied Biosystems) automated sequencer. Genotype scoring was performed using STRand (https://vgl.ucdavis.edu/informatics/strand.php). It was assumed that a single panmictic population was being assessed^31^, and downstream analyses of the 2013 sample pooled all locations into a single year population. To filter for missing data, the *missingno* function of the poppr^45^ R-package^46^ was used, removing loci with more than 25%, and individuals with more than 10% missing data, respectively. Genotyping errors and null alleles were estimated using calculations by Summers and Amos^47^, Dempster et al.^48^, Brookfield^49^, and Chakraborty et al.^50^.

### Genetic diversity

Calculations of observed (*H*_*o*_) and expected (*H*_*e*_) heterozygosity, allelic richness (*Ar*) and the inbreeding coefficient (*G*_*is*_) were made for each locus and across all loci to assess changes in genetic diversity. These statistics were computed using diveRsity package in R^46,51^. *H*_*e*_ differences between 2001 and 2013 were calculated using a Monte Carlo exact test based on 10,000 permutations from Adegenet^52^. Allelic richness was calculated with a 1000 re-sampling rarefaction technique with replacement using the smallest sample size in the data set to standardise for differing sample sizes (i.e. 2001 sample with 33 successfully genotyped individuals). A measure of private allelic richness (*PAr*) was also calculated by HP-Rare 1.1^53^.

### Relatedness analysis

Relationships between individuals from the two sampling years were estimated with the program ML-RELATE^54^. ML-RELATE calculates coefficients of relatedness (r) and putative relationships among individuals (e.g., unrelated, siblings, parent/offspring) using a maximum likelihood approach. Random genotype simulations were performed 1000 times for likelihood ratio tests and identified plausible relationships (full-sibling, half-sibling, parent-offspring, unrelated) based on a 99% confidence interval criterion^54^. Estimates of the percentage of sample pairs identified as full-siblings and half-siblings were calculated. ML-RELATE can accommodate the presence of null alleles in the estimates via a Monte-Carlo randomization test^55^ and the U-statistic of Rousset^56^. The test is one-tailed, i.e. it estimates the probability of obtaining the observed U statistic or greater value under Hardy–Weinberg conditions. If null alleles are present, ML-RELATE will use a maximum likelihood estimate of their frequency in all calculations which is considered to be more accurate than other estimators^47,49,50,54^.

### Inbreeding

Estimates of inbreeding depression often rely on limited datasets that can be affected by the presence of null alleles, which have been shown to induce an upward bias in inbreeding coefficient (*F*_*is*_) estimates^57^. This is especially true when sampling in small geographical areas in which inbred or closely related individuals occur^58,59^, as is the case for *H. guttulatus* from Ria Formosa. Therefore, in this study, simultaneous estimation of the inbreeding coefficient, null allele frequencies and random genotyping failures were conducted using the software INEST v2.2^59^. This software implements a Bayesian approach comparing different combinations of parameters (f = inbreeding, n = null alleles, b = genotype failures) and chooses the model with the lowest deviance information criterion (DIC) as the best fit. MCMC (Monte Carlo Markov Chain) used 500,000 cycles and a burn-in of 50,000, retaining parameters every 100th cycle.

### Genetic demography

To explore the possibility of a demographic bottleneck in Ria Formosa seahorses, two tests were performed. First, we used a test for heterozygosity excess^60^ implemented in INEST v2.2^59^, under the null expectation that a population under a mutation-drift equilibrium should have an equal probability of exhibiting a heterozygosity excess or deficiency at a given locus. Populations that have experienced a recent reduction in size typically exhibit fewer alleles and lower heterozygosity. However, allelic diversity declines at a faster rate than heterozygosity due to random genetic drift, which eliminates lower frequency alleles. Thus, this test contrasts the expected heterozygosity assuming Hardy–Weinberg equilibrium with expected heterozygosity under mutation-drift. The latter is more sensitive to declines in low-frequency alleles that are expected when populations become reduced in size. A bottleneck is inferred when heterozygosity under Hardy–Weinberg equilibrium exceeds heterozygosity under mutation-drift^60,61^. This analysis was run using a two-phase mutation model (TPM) with the proportions of multi-step mutations (*p*_*g*_*)* set as 0.22 and the mean size of multi-step mutations (*D*_*g*_) as 3.1 as recommended by Peery^61^. Stepwise mutation (SMM) and infinite allele models (IAM) were also carried out for comparison, as these represent the extremes of mutation models. The Wilcoxon signed-rank test was applied to test for the significance of heterozygosity excess for both mutation models using 10^6^ permutations to approximate the exact value, as well as combined z-scores^62^.

Second, a mode shift test was used to determine whether a shift has occurred in allele frequency classes. This test assumes that stable populations have a peak in allele numbers at the lowest frequency class, displaying an L-shaped distribution. Population bottlenecks tend to distort this distribution to the right, as the loss of alleles results in a rightward skew in the allelic distribution^62^. To test if the data conformed to the L-shaped null hypothesis of a stable population, Bottleneck v2.2.01^63^ was used.

### Effective population size

Estimates of contemporary effective population size (*N*_*e*_) may be obtained from single or temporal samples^64^. In this study, the former approach was used, because the samples from 2001 and 2013 covered a restricted time interval and there were no age estimates from the sampled individuals^65,66^. *N*_*e*_ was assessed from levels of linkage-disequilibrium (LD)^67^ with NeEstimator v2.1^68^. Monogamy was assumed^69^ and 95% confidence intervals of *Ne* were estimated using the jackknife‐across samples method^70^. Sample sizes are an important consideration when estimating *N*_e,_ and small sample sizes can result in considerable biases^66^. Due to the sample size disparity between the 2001 and 2013 samples, genetic data for both years were rarefied by generating 1000 replicate files with 30 individuals in each. Alleles with low frequencies (< 0.02) were omitted to avoid bias because their inclusion can bias estimates of *N*_*e*_^68^. Population-specific *N*_*e*_ was calculated by taking the harmonic mean of LDNe estimates from each year the population was sampled. As 95% confidence intervals of Ne based on the LD often included infinity, only 95% lower bounds are reported, as these are considered valuable indicators of changes in population size^64^.

## Results

A total of 434 individuals were screened across 12 loci (https://tinyurl.com/ya5tgk6a; Annex B). Four loci (*Hgut9, USC1, USC6* and *USC7*) and 135 genotypes were removed after filtering at 25% and 10% of missing data, respectively, resulting in 291 informative individuals across eight loci, 33 individuals in 2001 and 258 in 2013. Estimated null allele frequencies averaged across loci were low (< 9%) for both years, except for *Hhip3* locus from the 2001 sample, ranging between 0.147 and 0.198 across all four methods. Samples from 2001 show a higher frequency of null alleles (< 9%) than in 2013 (< 3%), possibly due to the poor preservation of the older samples.

### Between-year comparative genetic diversity

**Figure 3:**
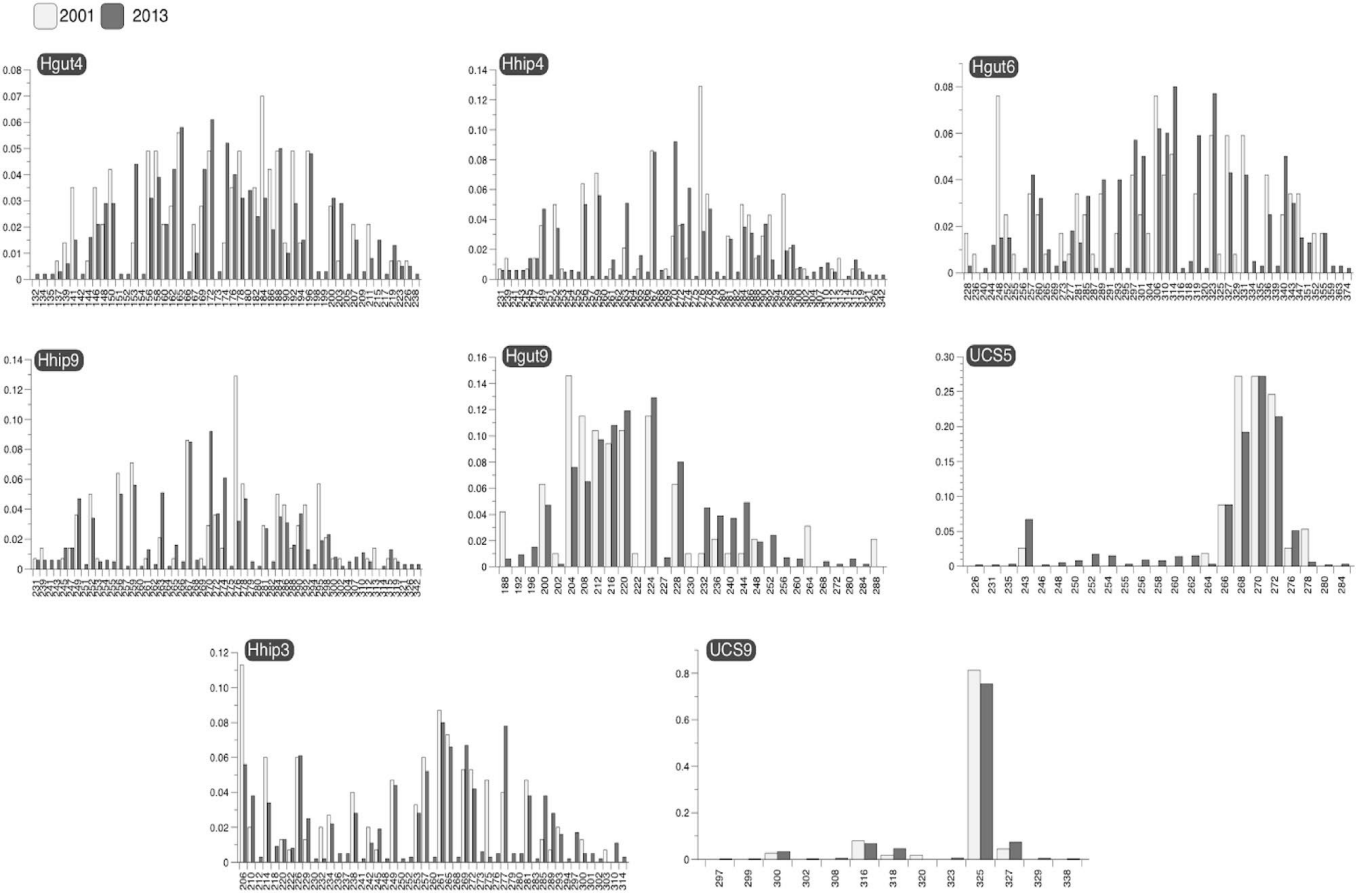
Allele frequencies of eight microsatellite loci in *H. guttulatus* from Ria Formosa.

The number of alleles per locus ranged from 6 to 28 in 2001 and from 12 to 51 in 2013 (Table 1), with a total of 150 and 292 alleles of variable frequency, respectively (Fig. 3). Average observed (*H*_*o*_) and expected (*H*_*e*_) heterozygosity was over 0.72, showing an overall increase in time (Table 1). The increase in He between 2001 and 2013 was supported by a significant Fis, which measures the reduction of heterozygosity of a population due to inbreeding via a Monte Carlo-based test (2001: 0.142, 95% CI:0.102–0.190; 2013: 0.049, 95% CI:0.031–0.067). Allelic richness averaged across loci was also significantly different between years (paired t-test; *p* < 0.05), increasing from 15.8 in 2001 to 20.3 in 2013 (Table 1), and average *PAr* increased from 4.1 to 6.5 during this period (Table 1), despite a decrease at the *Hgut6* locus from 8 to 4. The average inbreeding coefficient across loci declined from 0.16 in 2001 to 0.05 in 2013 (Table 1).

**Table 1:** Diversity indices for the two sampling periods (2001 and 2013).

Locus	*Na*		*Ar* = 30		*PAr*		*H*_*o*_		*H*_*e*_		*G*_*is*_	
	2001	2013	2001	2013	2001	2013	2001	2013	2001	2013	2001	2013
*Hgut4*	27	47	23	28	5	7	0.88	0.93	0.97	0.97	0.09	0.03
*Hgut6*	28	42	23	23	8	4	0.85	0.92	0.96	0.96	0.12	0.04
*Hhip1*	20	43	18	24	4	10	0.91	0.92	0.95	0.96	0.04	0.04
*Hhip3*	16	44	14	22	3	10	0.61	0.89	0.92	0.95	0.34	0.06
*Hhip4*	21	30	18	21	3	4	0.82	0.95	0.95	0.95	0.14	0.00
*Hhip9*	24	51	19	26	6	10	0.79	0.85	0.94	0.96	0.16	0.12
*USC5*	8	23	7	11	1	5	0.64	0.76	0.80	0.82	0.20	0.08
*USC9*	6	12	5	6	1	2	0.27	0.41	0.35	0.42	0.22	0.04
Average	19	37	16	20	4	6	0.72	0.83	0.85	0.87	0.16	0.05

*Na* number of alleles; *Ar* allele richness rarefied to the smaller *N*; *PAr* private allelic richness (unrarified); *H*_o_ observed heterozygosity; *H*_*e*_ expected heterozygosity; *G*_*is*_ inbreeding coefficient.

### Relatedness analysis

The analysis of genetic relationships between individuals, with or without the accommodation for the presence of null alleles, did not change the results significantly (*p* > 0.05). Inference of all 528 pairwise relationships for 33 individuals from 2001 identified 22 (4%) half-sibling pairs. Of the 31,878 pairwise relationships between the 258 individuals collected in 2013, ML RELATE classified 2482 (8%) as half-sibling pairs and 62 (0.2%) as full-sibling pairs.

### Inbreeding

The Bayesian approach of simultaneous examination of null alleles and inbreeding indicated the best model for 2001, according to DIC values, to include only the inbreeding (f) parameter (Table 2). However, the difference in DIC values between the model incorporating only inbreeding and the model containing null alleles (fn ΔDIC = 0.4) is modest, as DIC values are as large as 21,000. The most parsimonious model (f) has Avg (Fi) values of 0.14, similar, but slightly lower than *G*_*is*_ estimates (Table 1), whereas, the model including null alleles (fn) results in Avg (Fi) of 0.09. Regardless of the model choice, 95% HPD values of both these models do not include zero, indicating low to mild inbreeding. Using the 2013 data, the best fit model includes inbreeding and random genotyping failures (fb). However, this fit is similar, and only slightly better than the f, fn and fnb models, as all ΔDIC values are ≤ 1.2 (Table 2). Such a small degree of improvement in DIC values from the full model (fnb) to the model with the lowest DIC (fb) (ΔDIC = 1.2) suggests that the most appropriate model includes all parameters. The mean inbreeding coefficient of the optimal model was low (Avg (Fi) = 0.053; 95% HPD = 0.0412–0.066) and the 95% HPD does not include zero, consistent with minimal inbreeding in the sample.

**Table 2:** INEST best Bayesian model to estimate inbreeding coefficients adjusted for possible null alleles for 2001 and 2013 samples.

Model	2001				2013				95% HPD
	DIC	Δ DIC	Avg (Fi)	95% HPD	Model	DIC	ΔDIC	Avg (Fi)	
f	21,474.7	0.0	0.141	0.0941–0.2001	fb	21,474.8	0.0	0.053	0.0417–0.0662
fb	21,474.8	0.1	0.142	0.0916–0.1996	f	21,474.9	0.1	0.053	0.0409–0.0657
fn	21,475.2	0.4	0.085	0.0004–0.1641	fn	21,475.5	0.6	0.034	0.0102–0.0556
fnb	21,478.2	3.5	0.084	0.0000–0.1607	fnb	21,476.0	1.2	0.034	0.0108–0.0557
nb	21,482.2	7.4	NA	NA	n	21,484.9	10.1	NA	NA
n	21,482.3	7.5	NA	NA	nb	21,485.2	10.4	NA	NA
Null	21,615.6	140.9	NA	NA	Null	21,615.6	140.8	NA	NA
b	21,617.3	142.6	NA	NA	b	21,616.9	142.0	NA	NA

Models abbreviations: *f* inbreeding, *n* null alleles, *b* genotyping errors, *fnb* combinations of models f, n, and b, and *null* the null model; *DIC* Deviance Information Criterion, *Δ DIC* change in DIC value between models, *Avg (Fi)* sample mean inbreeding coefficient, *95% HPD* high and low posterior density interval.

### Genetic demography

Overall, both genetic bottleneck analyses do not reflect the drastic decrease observed in the census data. From the eight loci examined, the results from the TPM were not significant (*p* > 0.05) for heterozygosity excess for either the Wilcoxon signed-rank test (*p* = 0.96) or the *z*-test for combined *z*-scores (*p* = 1). Similar non-significant results (*p* > 0.05) were obtained for SMM and IAM, which represent opposite ends of the mutation spectrum (SMM Wilcoxon’s test *p* = 0.99 and *z*-test *p* = 1; IAM Wilcoxon’s test *p* = 1 and *z*-test *p* = 1). All loci except two (*Hgut4* and *Hhip4*) showed a deficiency of heterozygotes. Similarly, no distortion to the typically L-shaped distribution of allele frequencies was found (98% of alleles occurring in < 0.1 frequency class), consistent with a stable population size.

### Effective population size

**Figure 4:**
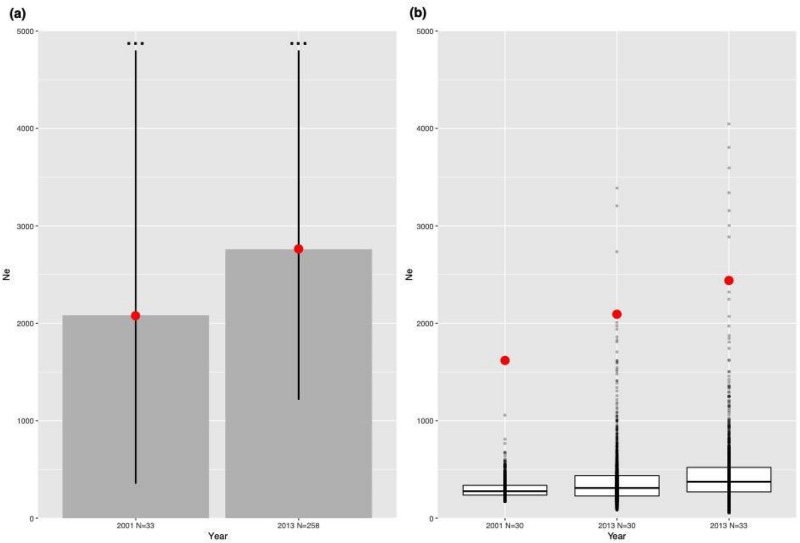
Effective population size (*N*_*e*_) estimates determined using the linkage disequilibrium method with a minimum allele frequency of 0.02. (**a**) Point estimates for 2001 (N = 33) and 2013 (N = 258). Error bars indicate 95% confidence intervals estimated by jackknife estimates, where those running off the chart include infinity. Red dots represent point estimates. (**b**) Lower bound values of the confidence intervals for 2001 (N = 30) [the reduction from 33 to 30 is to allow resampling] and 2013 (N = 33) from 1000 re-sampled files. Red dots represent harmonic means of all the point estimates based on 1000 replicates.

The results from both 2001 and 2013 datasets revealed *N*_*e*_ values of the same order of magnitude (Fig. 4a,b) for both tested methods. Both approaches (single point estimate with true sample size values, and point estimates using randomly generated files with rarefied sample sizes) provided larger estimates for the 2013 sample. However, the 95% confidence intervals for population-specific *N*_*e*_ values overlapped, and upper confidence limits were indeterminate (Fig. 4a).

## Discussion

The initial hypothesis for this investigation was based on the premise that the drastic decline in census population numbers reported between 2001 and 2008 would be reflected in reduced genetic diversity in subsequent generations. Based on this prediction, lower genetic diversity was expected in the 2013 sample. In contrast to this expectation, the *H. guttulatus* population of the Ria Formosa showed an increasing level of genetic diversity between 2001 and 2013. Only the number of private alleles at one microsatellite locus (*Hgut6)* showed any substantial decrease. Over this same period, although *N*_*e*_ has risen and *F*_*is*_ has seemingly declined, no evidence was found to support the occurrence of a genetic bottleneck, apart from the observation that the overall relatedness has increased. Before discussing the implications of these findings, it is important to highlight some of the caveats of this study.

Firstly, it must be stressed that the sample size disparity between both years may have, to some extent, influenced the results presented here. Some diversity estimates are more prone to biases due to sample size than others, making them more useful in the context of a comparison between years. For instance, while the number of alleles per locus in a small sample is greatly biased relative to an estimate based on a large sample size, this can be overcome with rarefaction, which improves the ability to compare between samples differing in size^71^. Secondly, the genetic diversity of the 2001 sample was derived from a higher number of sites (15) than that sampled in 2013 (6)^26,36^. Thirdly, the presence of null alleles in the dataset may have affected the study, particularly as cross-species amplification was used^72^. While the frequency of null alleles in the 2001 dataset was higher than that inferred for 2013, likely due to poor preservation, no evidence of null alleles in previous studies has been detected using these markers^43,44^. Thus, we found no reason to alter allele frequencies or exclude loci, except when using INEST, in cases in which the inclusion of null alleles or genotyping errors was found to upwardly bias *F*_*is*_ estimates. Finally, it is important to note that neutral microsatellites, particularly a limited number of loci, do not represent genome-wide diversity, and may fail to detect changes in the genetic diversity of loci experiencing selection^73^.

### Genetic diversity

Genetic diversity indices were generally high and showed an overall increase between 2001 and 2013, rejecting our initial hypothesis of a reduction in genetic diversity due to census population declines over the study period. The genetic diversity observed in our study averaged across loci (*Na*: 19/37; Ar(30): 16/20; *H*_*e*_: 0.85/0.87 for 2001 and 2013, respectively) is higher than that detected in Ria Formosa samples from 2004/2005 with values averaged across five sampling sites of *H*_*e*_: 0.37; *H*_*o*_: 0.36^74^. Although these differences may be due in part to the use of different markers, *Hgut4* was used in both studies, and as both studies employed similarly large sample sizes, this is unlikely to be a sampling artefact. When all available demographic and genetic diversity results are taken into consideration (Table 3), evidence for an association between population reductions based on census data and reductions in genetic diversity as estimated by microsatellites is equivocal. In other similar lagoonal environments in the Mediterranean, Taranto and Thau, expected heterozygosities at eight microsatellites loci are very low (*N*: 40, *H*_*e*_: 0.31 and *N*: 8, He: 0.39, respectively)^34^. Other coastal locations in the Mediterranean typically display low expected heterozygosities ranging from 0.30 to 0.54 with sample sizes from 2 to 17 see Table 3^34^. In *H. guttulatus* from the Galician coasts in NW Spain, 32 individuals scored for 12 microsatellite loci, yielded *H*_*e*_ of 0.500^43^. Another study with samples from the same area, but from a considerably larger number of individuals, 255 and 13 microsatellite loci, resulted in overall expected heterozygosities (observed heterozygosities values not reported) lower than 0.620^33^. However, common to our study are four loci, Hgut4, Hgut6, Hgu-USC5 and Hgu-USC9. Except Hgu-USC9 which has very low heterozygosity (ranging from 0.29 to 0.40) the remaining loci display high expected heterozygosities (ranging from 0.77 to 0.97), in line with the present study (Hgut4, Hgut6, Hgu-USC5 heterozygosities ranging from 0.80 to 0.97, and Hgu-USC9 from 0.35 to 0.40). Therefore, in spite of the severe population decline, Ria Formosa displays higher heterozygosities than coastal or lagoon populations elsewhere in Europe.

**Table 3:** Summary results from previously published genetic and demographic data from Ria Formosa.

Year	Demography	Marker	N	Allele numbers (average)	Genetic diversity	*Fis*	Bottleneck detected	Reference for genetic data
2001	High	Microsatellites	76	19	0.72	0.16	No	This study
2004/2005	High	mtDNA	29	11	0.72–0.88	0.032–0.102	Not tested	Woodall^74^
2008/2009	Low	Microsatellites	50	7.6	0.36	0.04	Inferred	Woodall et al.^32^
2012	High	No data						
2013	High	SNP	38	No data	No data	− 0.014	Not tested	Riquet et al.^31^
2013	Low	Microsatellites	258	37	0.83	0.05	No	This study

*N* number of individuals analysed, genetic diversity: observed heterozygosity, for microsatellite data, and haplotype diversity for mtDNA data.

The apparent reduction in *H*_*e*_ and *H*_*o*_ between 2001 and 2004/2005^74^ samples is perplexing, considering that this time interval is only equivalent to approximately two generations^23^. A possible scenario is that gene flow into the Ria Formosa has enabled some level of genetic diversity to permeate back into the population, a pattern supported by a more extensive temporal survey of seahorses in the area (Wilson et al*.*, unpublished data). This hypothesis is also supported by studies that have shown that South Iberian populations display low levels of microsatellite genetic differentiation^74^ and are panmictic with respect to 286 SNPs^31^. Despite suggestions that seahorses are capable of assisted long-distance translocations, likely through rafting on floating debris, carried by oceanic and coastal currents^75^, that this is implausible in the Ria Formosa, where *H. guttulatus* have high site-fidelity and very limited dispersal capacity^38^, and the lack of intermediate required habitat does not facilitate seahorse dispersal. The panmictic nature of the South Iberian populations remains to be fully explained even though a commonly cited rule of thumb is that one migrant per generation is needed to sufficiently minimise the loss of polymorphism and heterozygosity within subpopulations^18,76,77^.

### Relatedness and inbreeding

While the present results are inconsistent with a genetic bottleneck related to the inferred population decline, heterozygote deficiency was noted at six out of the eight loci, which could be a result of recent population expansion^60^, corroborating other findings^36,37^. It is possible that the doubling of related individuals between 2001 and 2013 is an artefact of the demographic decline inferred in 2008. With fewer individuals in the period after the population reduction, the likelihood of sampling related individuals would be expected to be higher, due to the remaining individuals contributing to the recovery reported by 2012. Nevertheless, both our investigation and Woodall’s^74^study used the heterozygosity excess method^60^, which is expected to have more power to detect recent population bottlenecks relative to the M-ratio statistic^78^. With this in mind, we suggest that *H. guttulatus* in the Ria Formosa may be naturally prone to population fluctuations that may be exacerbated by anthropogenic activity, e.g. clam farming, fisheries, dredging^79,80^. Seahorse populations are vulnerable to fluctuations^81–83^, which makes the conservation of their preferred habitats critical^26,41^ and of great urgency if disturbances and habitat degradation are identified.

### Effective population size

Both 2001 and 2013 *N*_*e*_ point estimates were negative, indicating that the results can be entirely explained by sampling error rather than drift^68^. This situation usually occurs when *N*_*e*_ is large, which may suggest that the Ria Formosa population was substantial during both sampling periods. Linkage disequilibrium due to drift is low in large populations and difficult to detect, rendering the estimation of *N*_*e*_ in large populations (i.e., *N*_*e*_ > 1,000) challenging, particularly with the small number of markers employed in this study^64^. More comprehensive genetic screening via high-throughput sequencing may offer an opportunity to overcome this limitation, and more precisely estimate *N*_*e*_^64^. Reporting the lower limits of these estimates remains valuable as an indicator of changes in population size^64^. Here, lower bound *N*_*e*_ means remained relatively stable, with a slight increase, less than the increases inferred from harmonic means and point estimates. The interpretation of these *N*_*e*_ estimates must be made with caution^84^ because there is only weak genetic differentiation between the Ria Formosa and other South Iberian subpopulations^31,74^. This implies a level of historical and/or contemporary gene flow, which seems improbable given the species’ life-history traits and habitat preferences. Nevertheless, *N*_*e*_ estimates may be inflated and/or reflect Ne of a larger metapopulation rather than the local population. While this cannot be ruled out, even with an apparent increase in *N*_*e*_, the estimates of harmonic means reported here centre around 2000 individuals, and 50% of the lower bounds are below 500 (Fig. 4b). A commonly used guideline employed in a conservation context is the 50/500 *N*_e_ rule of thumb^85^, although more recently, this has been revised upward to 100/1000 *N*_e_^86,87^. *N*_*e*_ ≥ 100 thought to be required to avoid inbreeding depression and limit the loss of total fitness to 10% and *N*_*e*_ ≥ 1000 is suggested in order to retain evolutionary potential in perpetuity. Our lower bound *N*_*e*_ estimates are worryingly close to levels thought to be required for the maintenance of long-term evolutionary potential (500/1000 *N*_*e*_), particularly in light of unpublished data that has shown that another severe decline has taken place, indicating an overall decline over the last 20 years.

Overall, based on the results presented here and in previous literature, it seems that the *H. guttulatus* population of the Ria Formosa has been able to retain relatively high genetic diversity despite evidence of a recent demographic decline. Perhaps the decline reported in 2008 was not sustained for long enough for drift to have had an effect and a sufficient number of individuals were left to retain high genetic diversity. According to empirical observations e.g. kit foxes^88^ and simulations^89^, changes in genetic diversity following a decline in population size may take a number of generations to become obvious, and that the short time span of our study was not enough to capture the full range of genetic parameter fluctuations.

Future studies using an increased number of loci compared to those used here (such as Single Nucleotide Polymorphisms, SNPs) could help to test this prediction. Alternatively, a recently published survey of available seahorse microsatellites identified 18 highly polymorphic loci for *H. guttulatus*, which would provide a more extensive panel of markers for the analysis of neutral diversity^90^. If SNPs or more microsatellite loci reveal declines in genome-wide genetic diversity, *F*_*is*_ will become extremely critical, as small differences in *F*_*is*_ values can significantly alter extinction risk^18,91^. Furthermore, the estimation of *N*_*e*_ for the broader region may highlight if the *N*_*e*_ value reported here is inflated through gene flow. Considering the lower bound estimates of *N*_*e*_, the precautionary principle must be taken. We recommend that active management actions for this population should be continued and enhanced to protect the diversity and numbers indicated here.

Coastal lagoons such Ria Formosa rank among the most productive ecosystems on Earth, and provide a wide range of ecosystem services and resources. Anthropogenic impacts are escalating in many coastal lagoons worldwide because of increasing population growth and associated land-use alteration in adjoining coastal watersheds^92^. Among the main stressors affecting coastal lagoons are habitat loss and alteration; eutrophication; sewage and organic wastes; fisheries overexploitation; sea-level rise; chemical contaminants, sediment input/ turbidity and floatables and debris^93^. Despite Ria Formosa being a semi-protected lagoon, many activities that alter the environment are permitted, including legal fisheries, anchoring and dredging, which contribute to habitat loss in the lagoon^79,94^. Habitat loss was identified as the leading probable cause for seahorse decline^38^, which was to some extent confirmed by other studies^36,37^. The observed extreme fluctuations in seahorse populations are correlated to the availability of holdfasts^95^. However, we cannot discard the impact that other undetermined abiotic or biotic factors such as warmer temperatures, food availability, or abundance of predators^36^ or overfishing may have on local populations. There is empirical evidence of a rapid and somewhat uncontrollable unreported and unregulated fishing (IUU) activity which may be a significant factor contributing to population decline (J. Palma, person.comm.).

It is difficult to explain the maintenance of the high levels of genetic diversity observed in Ria Formosa individuals when census data suggest such a dramatic decline. While we cannot discard the possibility that the individuals have moved to other areas, the lagoon has been visited regularly by divers experienced in seahorse detection. During 2020, the number of visited sites was increased, and although an overall decline in population size was observed, local increases were found at the microhabitat level. Expansions of individual home ranges in response to low densities of potential mates have been observed in previous years^35^, suggesting that short distance migrations outside the survey area may have influenced census results. Seahorses have been found to colonize previously degraded habitats after being enriched with artificial structures^95^. We discard the possibility that the seahorses might have moved outside Ria Formosa in search of partners, because of the lack of suitable habitats (e.g., availability of holdfasts), both to the west and the east of the lagoon. It is perplexing not to find a genetic signature of the demographic bottleneck in terms of allelic loss and of heterozygote decrease. At this short time scale, it would be expected to see an effect in allele number if not a reduction in heterozygosities, as it is known that heterozygosity decay trails the allele number reduction^96^.

### Conservation implications

This study showed that genetic diversity and effective population size of the Ria Formosa long-snouted seahorse was retained through a severe recent demographic decline as inferred from census data. However, this population has suffered an even steeper decline in recent years (2018–2020) (J. Palma, pers. comm.). Therefore, despite the genetic results, conservation measures and demographic and genetic monitoring should continue to be implemented. Enhanced protection and restoration of *H. guttulatus* habitats would help to retain the genetic diversity in the Ria Formosa. The use of artificial holdfasts has been shown to be a useful tool for repopulating areas in which habitats have been damaged or disappeared^95^. However, this does not resolve the issue of degrading habitat, which should be at the forefront of conservation in the Ria Formosa. Clam farming poses a significant threat to the seagrass *Zostera noltii*^80^, and other species of seagrass have been degraded by coastal construction and dredging^79^. *H. guttulatus* have a preference for complex habitats^26,35,41^, which are likely to be subject to human impacts, fluctuations and disturbances in the dynamic barrier island system that characterizes the Ria Formosa^97^. Identifying temporally stable areas and enhancing habitat may help to ensure the long-term viability of seahorses in the Ria Formosa^98^, in addition to the enhanced protection of important refuges. Finally, future studies will need to clarify the potential impact of gene flow between subpopulations of the South Iberian Peninsula, as previously suggested by other authors^31,74^ in order to determine the relevant conservation unit for seahorses in the region.

## Conclusion

Molecular data suggest that, contrary to our initial hypothesis, a severe demographic decline reported in 2008 did not reduce the genetic diversity of *H. guttulatus* in the Ria Formosa, nor cause a genetic bottleneck, and that the effective size of this population has instead modestly increased. A more in-depth genomic study could help to illuminate genetic changes at the whole genome level and identify genetic regions experiencing differential selection, which may be especially prone to population fluctuations. Finally, although the goal of this study was to assess genetic changes in diversity, it must be acknowledged that the effective conservation of essential habitat is critically important^99,100^. Bearing in mind the observed pattern of population fluctuations, the use of artificial holdfasts may alleviate pressures from environmental variation. Active and enhanced monitoring and conservation of key habitats should remain at the forefront of conservation efforts in nearshore environments such as the Ria Formosa.

## Supplementary Information

Supplementary Table.
