# Supplementary material for: The paradox of retained genetic diversity of Hippocampus guttulatus in the face of demographic decline
Source: Sci Rep. 2021 May 17;11:10434. doi: 10.1038/s41598-021-89708-0 (PMC8129090; doi:10.1038/s41598-021-89708-0)
Supplement: Supplementary file 1 — Supplementary Table. [file 41598_2021_89708_MOESM1_ESM.pdf]

Electronic supplement Table 1. Microsatellite loci used in the present study.

The paradox of retained genetic diversity of *Hippocampus guttulatus* in the face of demographic decline

Rupert Stacy

Jorge Palma

Miguel Correia

José Pedro Andrade

Rita Castilho

Corresponding author: rcastil@ualg.pt

Electronic supplement Table 1. Microsatellite loci used in the present study.

Locus	Reference	Genbank accession number	Primer forward (5' -> 3')	Primer reverse (3' -> 5')	Repeat structure
Hgut4	Vliet 2009	GQ148733	GCCGCATACACTGGACCGCATC	TGCGAGCTACGTGAGGGAGAACATC	(GATA)25
Hgut6	Vliet 2009	GQ148735	AGAGACGCCCAGTCCACACTGAA	GAGGAGCAGAATGGTGGAAGGGACA	(ATAG)28
Hgut9	Vliet 2009	GQ148738	AATCACTTCTCAATCAACCAATCT	ATGTGATCAATGAAGCCCAAAC	(ATAG)17
Hhip1	Vliet 2009	GQ148720	TGCAACAGGACACCCAAGGTAGCA	ATGAAAAAGCCAAAGCGTGGCGG	(ATCT)42
Hhip3	Vliet 2009	GQ148722	TGATGTTGCGTCTAAACAAGTGACA	GCCACTCTAAAATGTGCCTCAGAAC	(TCTA)22TCCA(TCTA)9
Hhip4	Vliet 2009	GQ148723	CATGTAAACAAACTCCAGGCATCG	AGGGTGATTCCATTTTATTGCGGG	(AGAT)30
Hhip9	Vliet 2009	GQ148728	TGACACATGCTGGCTATGTCCGT	GCCCCTGATTAACCTTGAACAACCTC	(GA)20TACAG(TAGC)4(TAGA)22
Hgu-USC1	Pardo 2006	DQ986275	GGATTTGACCCATTTCGATG	GTGTATTTGGCGCTGTTTGA	(TG)9
Hgu-USC5	Pardo 2006	DQ986279	GTGTGTTGGATTGCTGGATG	ATGACAAGTGCCTGAGCGTA	(TGCG)4(TG)6 C(GT)4A(TG)3
Hgu-USC6	Pardo 2006	DQ986280	CAGTCCCTGAAGCTATTCCTGT	AAGGACTTTGTGTTCACTTGC	(GT)12
Hgu-USC7	Pardo 2006	DQ986281	CAGAGCAGTGTACCCATTCG	TTTCACCGTCCATCTTCCTC	(GA)8A(AG)10
Hgu-USC9	Pardo 2006	DQ986283	TTGCAGAATGTGGCTGGATA	AGTGGAGGCTGACAGGGTAA	(TG)14
